# An Efficient Online Trajectory Generation Method Based on Kinodynamic Path Search and Trajectory Optimization for Human-Robot Interaction Safety

**DOI:** 10.3390/e24050653

**Published:** 2022-05-06

**Authors:** Hongyan Liu, Daokui Qu, Fang Xu, Zhenjun Du, Kai Jia, Mingmin Liu

**Affiliations:** 1 State Key Laboratory of Robotics, Shenyang Institute of Automation, Chinese Academy of Sciences, Shenyang 110016, China; dkqu@siasun.com (D.Q.); xufang@siasun.com (F.X.); jiakai@siasun.com (K.J.); 2 Institutes for Robotics and Intelligent Manufacturing, Chinese Academy of Sciences, Shenyang 110169, China; 3 University of Chinese Academy of Sciences, Beijing 100049, China; 4 SIASUN Robot & Automation Co., Ltd., Shenyang 110169, China; duzhenjun@siasun.com (Z.D.); liumingmin@siasun.com (M.L.)

**Keywords:** human-robot interaction, kinodynamic path search, trajectory optimization, real-time collision avoidance, B-spline, replanning

## Abstract

With the rapid development of robot perception and planning technology, robots are gradually getting rid of fixed fences and working closely with humans in shared workspaces. The safety of human-robot coexistence has become critical. Traditional motion planning methods perform poorly in dynamic environments where obstacles motion is highly uncertain. In this paper, we propose an efficient online trajectory generation method to help manipulator autonomous planning in dynamic environments. Our approach starts with an efficient kinodynamic path search algorithm that considers the links constraints and finds a safe and feasible initial trajectory with minimal control effort and time. To increase the clearance between the trajectory and obstacles and improve the smoothness, a trajectory optimization method using the B-spline convex hull property is adopted to minimize the penalty of collision cost, smoothness, and dynamical feasibility. To avoid the collisions between the links and obstacles and the collisions of the links themselves, a constraint-relaxed links collision avoidance method is developed by solving a quadratic programming problem. Compared with the existing state-of-the-art planning method for dynamic environments and advanced trajectory optimization method, our method can generate a smoother, collision-free trajectory in less time with a higher success rate. Detailed simulation comparison experiments, as well as real-world experiments, are reported to verify the effectiveness of our method.

## 1. Introduction

Human-robot interactions have been involved in more and more applications, from automobile assembly to electronic product assembly applications [1,2]. In these applications, robots act as human partners, with side-by-side or face-to-face working with humans in shared workspaces to complete specific tasks, and the safety issue of human-robot coexistence becomes crucial [3,4]. For example, in the electronic product production line’s common robot pick-place tasks, the arms of human partners may appear on the predefined motion path of the robot. At this point, it may be inefficient and unsafe for the robot to simply back off or stop motion. Instead, it may make more sense to adjust online the performing trajectory of the manipulator to avoid potential collisions, while maintaining the original task undisturbed [5,6]. Therefore, an effective online trajectory generation method is urgently needed to help a manipulator perform autonomous motion in dynamic and uncertain environments. For trajectory generation in a dynamic environment, the trajectory needs to meet safety, smoothness, and dynamic feasibility requirements. In addition, since the movements of human partners or dynamic obstacles are highly uncertain, the trajectory generation process should also meet real-time requirements. To this end, a robust and efficient online trajectory generation method is designed to acquire a safe, smooth, and dynamically feasible trajectory in real time.

Many safety-related motion planning methods for dynamic environments have been developed at different control levels. The reactive planning method using the potential field concept has the advantages of low computational cost and high speed. Based on these characteristics, Flacco et al. [7] introduced a collision avoidance method based on the principle of infinite depth in the depth space. Nascimento et al. [8] proposed a safe contour collision avoidance method based on the finite depth principle by fusing an external vision sensor and robot body sensors information. Tulbure et al. [9] proposed a collision avoidance algorithm that maintains convergence to the goal by combining local reactive planning with global planning. Lin et al. [10,11] introduced a velocity-based physical human-robot interaction method for non-redundant robots, which allows the end-effector to track the human guidance trajectory while avoiding collisions of the links with obstacles. Different from the potential field method, the danger field is constructed based on the state of the robot itself rather than the state of obstacles [12], and indicates the degree of danger of the robot’s current position and velocity to objects. Based on the concept of danger field, Refs. [13,14] developed novel danger assessment and control methods for the safety of human-robot coexistence. Compared with the danger field, the safety field is generated using the obstacle state information and represents the source of danger in a more complete form [15]. Methods based on potential field and safety/danger field only consider the local space to avoid obstacles but may cause the robot to get stuck in local minima. Moreover, when the robot encounters obstacles, it may retreat from the original task instead of finding a new trajectory to reach the goal.

There are approaches that formulate trajectory planning as an optimization problem to generate collision-free trajectories. Zucker et al. [16] proposed a trajectory optimization method based on covariant Hamiltonian optimization (CHOMP), which iteratively optimizes a balance function including smoothness and collision costs to improve the initial trajectory quality. Schulman et al. [17] developed an efficient motion planning method that formulates a sequential convex optimization method and considers collision-free constraint formulae associated with obstacles. Zanchettin et al. [18] proposed a motion planning method by combining the trajectory planning method with the optimized control strategy. Ragaglia et al. [19] proposed a collision avoidance framework that takes safety requirements as constraints and maximizes production efficiency as an optimization goal. Most of these methods may generate collision-free trajectories with high computational costs and low success rates.

Learning-based planning methods have been developed for collision-free trajectory generation. Qureshi et al. [20] proposed a motion planning network (MPNet) that generates an end-to-end collision-free path from the starting point to the goal by encoding the workspace. However, this method does not consider dynamic obstacle constraints. Xu et al. [21] proposed a motion planning method based on a recurrent neural network. It defines a set of level set functions and virtual fences to encode the workspace to achieve obstacle avoidance and solves the quadratic programming problem online by a recurrent neural network to track the reference trajectory. Song et al. [22] proposed a robot trajectory planning method using a radial basis neural network to improve trajectory planning accuracy, but it does not consider obstacle avoidance. Shen et al. [23] proposed a redundant manipulator collision avoidance method by combining deep reinforcement learning and gradient projection methods. However, the real-world performance of this method has not been shown. Although great progress has been made in learning-based planning methods, considerable challenges remain in highly dynamic environments. Liu et al. [24] proposed a human-robot collaboration framework, which combined a human motion prediction model and a task model based on finite state machine to improve the efficiency of human-robot collaboration. It improves collaboration efficiency by predicting human motion trajectories, and the current work solves the problem of human-robot interaction safety via a hierarchical online trajectory generation algorithm. Our research method and objective are different from previous work.

Another class of methods used for motion planning in dynamic environments is replanning, which can update the robot motion trajectory according to the changes of dynamic obstacles in the workspace. To this end, Hauser et al. [25] proposed an adaptive time-stepping architecture for real-time replanning, which dynamically adapts the planning time and improves the stability of the replanner. Sun et al. [26] proposed a high-frequency replanning method with kinodynamic RRTs, which computes multiple RRTs in parallel and executes the first action of the optimal motion plan. Otte et al. [27] proposed a asymptotically optimal and single-query sampling-based replanning algorithm, which continuously improves and repairs the search graph during navigation to obtain the shortest path. Völz et al. [28] developed a predictive path following controller that time-parameterizes the planned path and computes optimal control actions along the planned path, and a continuous replanning strategy is invoked at fixed time intervals to avoid dynamic obstacles. Pupa et al. [29] proposed a safety-aware kinodynamic planning method, which replans the nominal trajectory by calling the RRT algorithm multiple times and scales the robot velocity according to safety rules. Most recently, Covic et al. [30] proposed a motion planning algorithm (DRGBT) dedicated to the fast exploration of dynamic environments by defining an adaptive horizon and a replanning mechanism. Sampling-based path-planning algorithms are used as initial path generators in these replanning algorithms. Sampling-based initial path generators are usually asymptotically optimal but computationally expensive. Moreover, the random behavior of sampling-based methods can also lead to unpredictable performance, especially with a limited number of samples [31]. Search-based methods play an important role in replanning due to the consistency of search results [32]. However, most of the existing search-based replanning methods [33,34,35] are aimed at robot systems with non-fixed bases, which may not be directly applicable to multi-joint collaborative robot systems. Table 1 summarizes and compares the similarities and differences of related works from five aspects: danger source; obstacle characterization; real-time performance; whether the collision avoidance trajectory tends to the target point (such as not simply retreating from the main task); and constraints.

Motivated by the above methods, this paper proposes a complete and efficient online trajectory generation method to help a manipulator for autonomous planning in highly dynamic environments. It does not suffer from expensive computational burdens or complex data structures and is suitable for real-time application. Our approach starts with an efficient kinodynamic path search method using heuristic search and linear quadratic minimum time control, which finds a safe, feasible, and time-minimized initial trajectory on voxel grids. Links constraints are introduced into the path search process to avoid invalid paths where links collide with obstacles. A trajectory optimization method using the B-spline convex hull property is adopted to post-optimize the initial trajectory to increase the clearance between the initial trajectory and obstacles and improve the smoothness. To avoid the collisions between the links and obstacles and the robot self-collision, a constraint-relaxed links collision avoidance method is designed by solving a standard quadratic programming problem, which minimizes the deviation between the actual trajectory and the back-end optimized trajectory. A derivative control point adjustment method is designed to eliminate infeasible higher-order derivatives. Finally, the path search module and the trajectory optimization module are integrated into a receding horizon replanning framework using a horizon-limited replanning mechanism.

Compared with the current state-of-the-art planning methods for fixed-base robots, the DRGBT algorithm and RRT*^X^* algorithm, and the advanced trajectory optimization methods, the CHOMP algorithm and the TrajOpt algorithm, our method can generate smoother, collision-free trajectories in shorter time with higher success rates. Numerous simulation experiments are performed to verify the effectiveness and robustness of the proposed method. Furthermore, in a real-world pick-place experiment, we also demonstrate that our method can effectively replan a new trajectory to avoid dynamic obstacles and converge to the item placement box. The contributions of this paper are summarized as follows:

(1) A complete and effective real-time online trajectory generation framework is developed bottom-up, which mainly includes kinodynamic path search, B-spline trajectory optimization, and links collision avoidance optimization.
(2) A path search method considering links constraints and a trajectory optimization method using the B-spline convex hull property are presented. The former transforms a position-only geometric search into an efficient kinodynamic search by state-space motion primitive generation and heuristic cost evaluation. The latter fully considers dynamic constraints and converges quickly to generate a safe, dynamically feasible, and smooth trajectory.
(3) A constraint-relaxed links collision avoidance optimization method is adopted, which effectively avoids link collisions while tracking the optimized task space trajectory.
(4) The proposed algorithm is deployed on a physical collaborative robot experimental platform. Detailed simulation comparison experiments and real-world experiments are performed to demonstrate the effectiveness of the proposed method.

The rest of this paper is organized as follows. The key points of the planning problem are stated in Section 2. Section 3 introduces the links-constrained kinodynamic path search method. Section 4 presents the trajectory optimization method. Section 5 elaborates on collision avoidance methods for both links and obstacles and robot self-collision, and the fusion process of links collision avoidance optimization and back-end trajectory optimization. Section 6 presents the experimental results. The conclusion is reported in Section 7.

## 2. Problem Statement

In this study, we solve an online trajectory generation problem for a manipulator in dynamic environments. Consider a human-robot collaboration application where an *n*-DOF manipulator runs from an initial configuration, $q_{s}$, to a specified goal configuration, $q_{g}$, and a dynamic obstacle, O, may appear on its motion path. Then, the robot’s trajectory, q(t), is considered to be feasible and collision-free when

(1) $$\begin{array}{cc} d\left(\chi_{lk}\left(q_{a}\right),O\right)\geq d_{0},d\left(\chi_{lk}\left(q_{a}\right),\chi_{li}\left(q_{a}\right)\right)\geq d_{l0} & \forall q_{a}\in q\left(t\right)\\ \; & \forall k\in \{1,\ldots ,n\},\forall i\in \{1,\ldots ,n\}\\ \; & \forall {\dot{q}}_{a}\in \left[\begin{array}{cc} {\dot{q}}_{\min} & {\dot{q}}_{\max} \end{array}\right]\\ \; & \forall {\ddot{q}}_{a}\in \left[\begin{array}{cc} {\ddot{q}}_{\min} & {\ddot{q}}_{\max} \end{array}\right] \end{array}$$

where $\chi_{lk}\left(q_{a}\right)$ represents the geometric line segment representation of the *k*-th link when the robot is in configuration $q_{a}$, and $\chi_{li}\left(q_{a}\right)$ is the geometric line segment representation of the links other than the *k*-th link and not adjacent to the *k*-th link. $d\left(\chi_{lk}\left(q_{a}\right),O\right)$ represents the minimum distance between the link segments and the obstacles. $d\left(\chi_{lk}\left(q_{a}\right),\chi_{li}\left(q_{a}\right)\right)$ represents the minimum distance between the *k*-th link and the *i*-th link. $d_{0}$ is the safe distance threshold between the links and obstacles, and $d_{l0}$ is the safe distance threshold of the self-collision. For this reason, the shared workspace is equipped with a monitoring system that detects obstacles and estimates the distance between obstacles and the robot. Several methods for real-time obstacle detection can be found in the literature [36,37,38].

**Figure 1:**
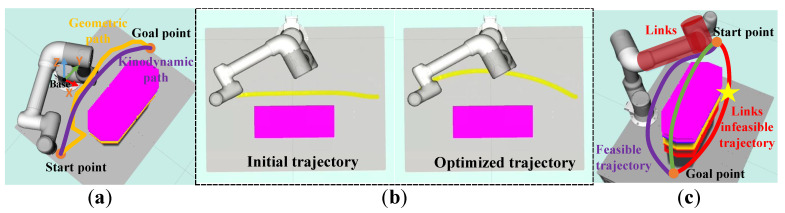
(**a**) The geometric and kinodynamic initial trajectories. (**b**) The initial and optimized trajectories. (**c**) Links-constrained task space trajectories. The red trajectory is collision-free for the end-effector, but the links may collide with the obstacle.

We aim to design an efficient trajectory generation method to solve the above problems. There are several key points that need to be addressed:

- A safe and feasible initial trajectory from the starting position to the goal needs to be searched. Traditional path-planning algorithms such as A*, Dijkstra, and the sampling-based method RRT usually do not consider the nonstatic initial state of the robot, so there are problems in replanning. As shown in Figure 1a, the geometric shortest path may turn sharply, which may lead to the failure of path parameterization. Since the replanning has non-general dynamic characteristics, it is necessary to use the kinodynamic planner to achieve a nonstatic initial state to ensure dynamic feasibility.
- Since the initial trajectory search does not consider the distance cost, the initial trajectory tends to be close to obstacles (see Figure 1b). In addition, the uncertainty of dynamic obstacle motion may also make the initial trajectory unfeasible. Therefore, trajectory optimization and replanning strategies are necessary.
- Collision avoidance of the robot links needs to be considered. As shown in Figure 1c, although the red trajectory indicated by “yellow star” is feasible for the end-effector, the robot links will collide with obstacles if the end-effector runs along with it.

## 3. Links-Constrained Kinodynamic Path Search

We propose an efficient links-constrained kinodynamic path search method for the path search of a fixed-base *n*-DOF manipulator in a dynamic environment. It fully considers the link constraints in the path search process and realizes an efficient kinodynamic search by applying a series of discrete control inputs. Different from the traditional A* algorithm, nodes are not searched along a straight line but use a set of short-duration motion primitives related to the robot state to generate the edges of the graph. In addition, unlike quadrotors and unmanned vehicles, whose bodies can move flexibly in three-dimensional space, multi-joint robots with fixed bases need to fully consider the constraints of obstacles on the links (see Figure 1c).

The path search process is summarized as Algorithm  1, and the mathematical principles of the key components of the search algorithm (motion primitive generation, search cost evaluation) are also explained in detail. We take the robot task space starting state and goal state, the safe distance threshold, and a set of discrete control inputs as input, and a safe, feasible, and time-optimal initial trajectory is searched as output. Following the A* algorithm, we denote open set and closed set as P and C, and current grid node as $p_{cur}$. Given the current state, discrete control inputs ***u***, and duration τ, the **NodesExpand**() function produces discrete motion primitives (see Section 3.1). Primitive nodes that end in the same grid with a non-minimum search cost will be removed. The interest points on the links corresponding to the node that satisfies the feasibility check needs to be collision detected by Equation (1). The positions of interest points on the links are obtained using the **Kinematicsmodel**() function, and the distance between interest points and obstacles are calculated using the euclidean distance in the **Distance**() function. Once the minimum distance between the interest point and the obstacle approaches the safe distance threshold $d_{0}$, the collision signal will be output by the **CollisionCheck**() function (see Section 5), and the new extended primitive node is marked as infeasible. The minimum search cost from the starting state to the current state is calculated by minimizing the time cost and control cost. Heuristic search method using Pontryagin’s minimum principle to minimize initial trajectory time (Section 3.2). The entire process continues until the robot successfully reaches the goal region.
 **Algorithm 1** Links-constrained Kinodynamic Path Search Algorithm**Input:** Inital and goal state: $x_{s}$, $x_{g}$; The safe distance threshold: $d_{0}$; Obstacle: O; Discretized control input sets *U***Output:** Time-optimal initial trajectory: Γ;  1:$p_{0}\leftarrow x_{s}$, $p_{g}\leftarrow x_{g}$;  2:P←⌀, C←⌀;  3:P.add($p_{0}$);  4:**while**P!=⌀ **do**  5:    $p_{cur}\leftarrow$P.**pop**(), C.**insert**($p_{cur}$);  6:    **if** $p_{cur}\in p_{g}^{near}$ **then**  7:        **return** Retrieved_Path;  8:    **end if**  9:    nodes←**NodesExpand**($p_{cur}$, U, τ);  10:  **for** $p_{i}$ **in** nodes **do**  11:        interest points← **Kinematicsmodel**($p_{i}$);  12:        min_dis←**Distance**(interestpoints, O);  13:        sig← **CollisionCheck**(min_dis, $d_{0}$);  14:        **if** (sig) **then**  15:           **Feasible**$\left(p_{i}\right)$←false;  16:        **end if**  17:        **if** $\left(p_{i}\notin C\right)$ ∧ **Feasible**$\left(p_{i}\right)$ **then**  18:           $g_{temp}\leftarrow p_{cur}.g_{c}$ + **EdgeCost**($p_{i}$);  19:           **if** $p_{i}\notin P$ **then**  20:               P.**add**($p_{i}$)  21:           **else if** $g_{temp}\geq p_{i}.g_{c}$ **then**  22:               continue;  23:           **end if**  24:           $p_{i}.parent\leftarrow p_{cur}$, $p_{i}.g_{c}\leftarrow g_{temp}$, $p_{i}.f_{c}\leftarrow p_{i}.g_{c}$ + **Heuristic**($p_{i}$);  25:        **end if**  26:    **end for**  27:**end while**

### 3.1. Motion Primitives for Node Expansion

We consider an environment with dynamic obstacles, where a robot with a fixed base and *n* joints moves its end-effector from an initial state $x_{s}$ to a goal state $x_{g}$. Let $p_{e}$ represent the end-effector position in the task space. The trajectory $p_{e}\left(t\right)$ executed by the end-effector can be decomposed of path-velocity decomposition [39],

(2) $$p_{e}\left(t\right)=p_{e}\left(s_{\mu }\left(t\right)\right),\;s_{\mu }\left(t\right)=\sum_{i=0}^{r}a_{r}t^{r}$$

where $s_{\mu }\left(t\right)$ is the time law of the given parametric geometric path $p_{e}\left(s\right)$, which is generated by a polynomial of degree *r*, $\mu \in \left\{x,y,z\right\}$. Let $x\left(t\right):={\left[p_{e}{\left(t\right)}^{T},{\dot{p}}_{e}{\left(t\right)}^{T},\cdots ,{p_{e}}^{\left(\kappa -1\right)}{\left(t\right)}^{T}\right]}^{T}$ be the state of a dynamic system consisting of a position and its κ−1 order derivative. Let $u\left(t\right)={p_{e}}^{\left(\kappa \right)}\left(t\right)$ be the control input and $u\left(t\right)\in U:={\left[-\mu_{\max},\;\mu_{\max}\right]}^{3}\subset R^{3}$. In this study, instead of using the control set U directly, we consider a lattice discretization $U_{d}\subset U$ following [40] for each axis, where each control vector $u_{d}\in R^{3}$ will define a short-duration motion for the system. The discretized $U_{d}$ is obtained by choosing a number of samples $\left\{-u_{\max},-\frac{\ell -1}{\ell }u_{\max},\cdots ,\frac{\ell -1}{l}u_{\max},u_{\max}\right\}$ along each axis with discrete steps $\nabla_{u}=\frac{u_{\max}}{\ell }$, and results in $M={\left(2\ell +1\right)}^{3}$ motion primitives [41]. A simple double integral system is considered for each axis (e.g., κ=2), and the discrete state space model can be defined as

(3) $$x_{d+1}=Ax_{d}+Bu_{d}$$

(4) $$A=\left[\begin{array}{cccccc} 1 & 0 & 0 & \tau & 0 & 0\\ 0 & 1 & 0 & 0 & \tau & 0\\ 0 & 0 & 1 & 0 & 0 & \tau \\ 0 & 0 & 0 & 1 & 0 & 0\\ 0 & 0 & 0 & 0 & 1 & 0\\ 0 & 0 & 0 & 0 & 0 & 1 \end{array}\right],\;B=\left[\begin{array}{ccc} 0 & 0 & 0\\ 0 & 0 & 0\\ 0 & 0 & 0\\ \tau & 0 & 0\\ 0 & \tau & 0\\ 0 & 0 & \tau \end{array}\right]$$

where τ represents the short-duration step. Finally, given the current state of the end-effector and a set of discrete control inputs, the motion primitives for node expansion can be obtained.

### 3.2. Search Cost Evaluation

The search cost from the starting node $x_{s}$ to the current node $x_{c}$ is calculated by minimizing the time cost and control cost, where the time cost ensures that the time is minimized, and the control cost makes the state trajectory as smooth as possible. The search cost function [40] is defined as follows,

(5) $$\min_{T}\;\;C\left(T\right)=\;\int_{0}^{T}{∥u\left(t\right)∥}^{2}dt+\rho T$$

where ${\int_{0}^{T}∥u\left(t\right)∥}^{2}dt$ indicates the smoothness of the trajectory, and ρ≥0 indicates the importance of the trajectory duration *T* relative to its smoothness. The cost of the motion primitive generated by the discrete control input $u{}_{d}$ and duration τ can be expressed as $g_{cd}=\left({∥u_{d}∥}^{2}+\rho \right)\tau$. Finally, the total search cost from the initial state $x_{s}$ to the current state $x_{c}$ is expressed as $g_{c}=\sum_{m=0}^{M}\left({∥u_{dm}∥}^{2}+\rho \right)\tau$.

The search cost from the current state $x_{c}$ to the goal state $x_{g}$ is obtained using a heuristic function, which is very useful for accelerating the search speed. Combined with Pontryagin’s minimum principle [42], the heuristic function is designed to solve the minimum time cost trajectory, that is,

(6) $$\begin{array}{cc} \; & s_{\mu }^{*}\left(t\right)=\left[\begin{array}{c} \frac{1}{6}\alpha_{\mu }t^{3}+\frac{1}{2}\beta_{\mu }t^{2}+v_{\mu c}t+p_{\mu c}\\ \frac{1}{2}\alpha_{\mu }t^{2}+\beta_{\mu }t+v_{\mu c} \end{array}\right],\\ \; & u^{*}\left(t\right)=\alpha_{\mu }t+\beta_{\mu } \end{array}$$

(7) $$\left[\begin{array}{c} \alpha_{\mu }\\ \beta_{\mu } \end{array}\right]=\left[\begin{array}{cc} -\frac{12}{T^{3}} & \frac{6}{T^{2}}\\ \frac{6}{T^{2}} & -\frac{2}{T} \end{array}\right]\left[\begin{array}{c} p_{\mu g}-p_{\mu c}-v_{\mu c}T\\ v_{\mu g}-v_{\mu c} \end{array}\right]$$

(8) $$C^{*}\left(T\right)=\sum_{\mu \in \{x,y,z\}}\left(\frac{1}{3}\alpha_{\mu }^{2}T^{3}+\alpha_{\mu }\beta_{\mu }T^{2}+\beta_{\mu }^{2}T\right)$$

where $p_{\mu c}$ and $p_{\mu g}$ represent the current and goal positions, respectively. $v_{\mu c}$ and $v_{\mu g}$ represent the current and goal velocities, respectively. $C^{*}\left(T\right)$ indicates the cost function. By solving the root $T_{f}$ of $\frac{\partial C^{*}\left(T\right)}{\partial T}=0$, the minimum time cost and feasible initial trajectory can be obtained. $C^{*}\left(T_{f}\right)$ indicates the minimum search cost [41]. Finally, the total search cost from the initial state to the goal state is denoted as $f_{c}=g_{c}+C^{*}\left(T_{f}\right)$.

## 4. Trajectory Optimization

Although the initial trajectory generated by the kinodynamic path search is dynamically feasible and collision-free, it may be suboptimal due to grids discretization and distance constraints with obstacles are not considered. In this section, the trajectory optimization method using the B-spline convex hull property is adopted to improve the smoothness of the trajectory and increase the clearance between the initial trajectory and obstacles. To adjust the infeasible high-order derivatives, an infeasible derivative control point adjustment method is designed using the derivative B-spline-bounded sufficient condition [43] and convex hull property. Furthermore, the planned initial trajectory may also become invalid as dynamic obstacles may appear at any time. To this end, a receding-horizon replanning strategy is utilized to generate a new trajectory to reach the goal.

### 4.1. B-Spline Curve Formulation

A B-spline curve c(t) [44] of degree *k* is defined by the linear combination of a set of control points $\left\{{\mathrm{cp}}_{0},{\mathrm{cp}}_{1},\cdots ,{\mathrm{cp}}_{n}\right\}$ and the B-spline basis functions $B_{i,k}$, i.e.,

(9) $$c\left(t\right)=\sum_{i=0}^{n}B_{i,k}\left(t\right){\mathrm{cp}}_{i}$$

where the B-spline basis functions $B_{i,k}$ can be computed by the fast and efficient de Boor algorithm [45] on a non-decreasing knot vector $\left\{t_{0},t_{1},\cdots ,t_{m}\right\}$. The number of knots, the degree, and the number of control points satisfy the relationship m=n+k+1. For a uniform B-spline, its knot vector is uniformly divided by $\Delta t=t_{m+1}-t_{m}$, where the *i*-th knot span is $\left[t_{i},t_{i+1}\right)$. In addition, each knot span is also normalized by $\xi =(t-t_{i})/\Delta t$ so that the knot vector covers the interval [0, 1]. On the *i*-th knot span $\left[t_{i},t_{i+1}\right)$, at most k+1 basis function is nonzero, namely, $B_{i-k,k}\left(u\right),B_{i-k+1,k}\left(u\right),\cdots ,B_{i,k}\left(u\right)$, corresponding to k+1 control points ${\mathrm{cp}}_{i-k},{\mathrm{cp}}_{i-k+1},...,{\mathrm{cp}}_{i}$. Furthermore, ${\mathrm{CP}}_{i-k}={\left[{\mathrm{cp}}_{i-k},{\mathrm{cp}}_{i-k+1},\cdots ,{\mathrm{cp}}_{i}\right]}^{T}$ is defined as the *s*-th control point span including k+1 continuous control points. The general matrix representation of B-spline is described in [46]. Then, the position and *l*th-order derivative [43] of the B-spline curve can be expressed as

(10) $$\begin{array}{cc} \; & c_{s}\left(\xi \right)=b^{⊤}M_{k}Y_{s}\\ \; & \frac{dc_{s}\left(\xi \right)}{d^{ld}\xi }=\frac{1}{{\left(\Delta t\right)}^{ld}}\frac{db^{⊤}}{d^{ld}\xi }M_{k}Y_{s} \end{array}$$

where

(11) b=1ξξ2⋯ξk⊤Mk=(mi,s)∈R(k+1)×(k+1)mi,s=1k!kk−i∑r=sk(−1)r−sk+1r−s(k−r)k−iYs=CPi−k

In addition, b is the basis vector, and $M_{k}$ denotes a blending matrix. When ld is equal to zero, the upper and lower equations of Equation (10) are equivalent.

### 4.2. Optimization Approach

**Figure 2:**
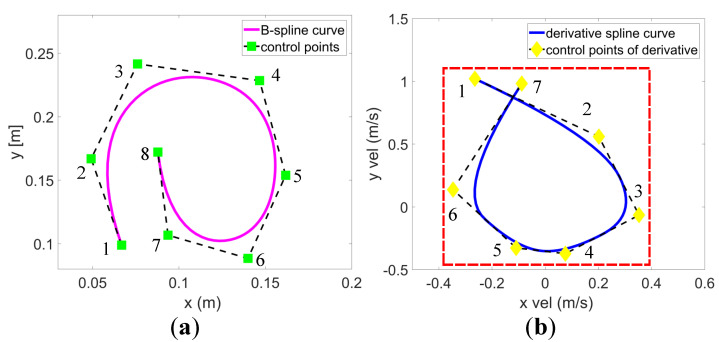
Illustration of the B-spline convex hull property. (**a**) Green square points indicate control points. (**b**) Yellow diamonds are derivative control points corresponding to the left image. The spline curves lie within the union of convex hulls of the control points. Moreover, the entire curve is feasible if all control points are within the feasible bounding box (red dashed box).

One of the important properties of B-spline is the strong convex hull property. As shown in Figure 2a, all points on a B-spline curve lie within the union of all convex hulls consisting of n+1 successive control points. More precisely, if $t\in \left[t_{i},t_{i+1}\right)$, then c(t) is in the convex hull of control points $\left\{{\mathrm{cp}}_{i-k},{\mathrm{cp}}_{i-k+1},{\mathrm{cp}}_{i}\right\}$. Another important property of B-spline is that the *d*-th derivative of a *k*-degree B-spline is a (k−d)-degree B-spline on the original knot vector with a new set of (n−d+1) control points $\left\{{\mathrm{cp}}_{0},{\mathrm{cp}}_{1},\cdots ,{\mathrm{cp}}_{n-d}\right\}$. For example, the velocity-spline curve shown in Figure 2b. Moreover, the control points of velocity $v_{i}\in \left\{[v_{min},v_{max}]\right\}$, acceleration $a_{i}\in \left\{[a_{min},a_{max}]\right\}$, and jerk $j_{i}\in \left\{[j_{min},j_{max}]\right\}$ are defined as

(12) $$\begin{array}{cc} \; & v_{i}=\frac{{\mathrm{cp}}_{i+1}-{\mathrm{cp}}_{i}}{\Delta t}\\ & a_{i}=\frac{v_{i+1}-v_{i}}{\Delta t}\\ & j_{i}=\frac{a_{i+1}-a_{i}}{\Delta t} \end{array}$$

where each knot span Δt is the same.

In the current work, three cost terms related to safety, smoothness, and feasibility are used to model the trajectory cost, where the safety term indicates the cost of approaching obstacles, the smooth term indicates the trajectory smoothness cost, and the feasible term indicates the dynamic feasibility cost. Then, the objective function is written as

(13) $$\min_{p}C=\varphi_{s}C_{s}+\varphi_{c}C_{c}+\varphi_{d}C_{d}$$

where $C_{s}$ represents smoothness cost, $C_{c}$ is collision cost, and $C_{d}$ is for feasibility cost. The weight coefficients $\varphi_{s}$, $\varphi_{c}$, and $\varphi_{d}$ trade off each cost term to minimize the final optimization cost.

(1) *Smoothness costs:* For the smoothness penalty term, an elastic band function is designed to describe the smoothness of the position control points, which only uses the geometric information of the control points without involving time information. Moreover, the smoothness of the B-spline trajectory can also be improved by minimizing the acceleration and jerk control points [47]. Then, the smooth term penalty function is defined as
  (14) $$C_{s}=\sum_{i=k-1}^{n-k+1}{∥\delta \left({\mathrm{cp}}_{i+1}-{\mathrm{cp}}_{i-1}\right)-\left({\mathrm{cp}}_{i}-{\mathrm{cp}}_{i-1}\right)∥}_{2}^{2}+\sum_{i=1}^{n-1}{∥a_{i}∥}_{2}^{2}+\sum_{i=1}^{n-2}{∥j_{i}∥}_{2}^{2}$$
  where the scaling factor $\delta =\frac{d_{i-1}}{d_{i-1}+d_{i}}$ ensures that the relative distance between two adjacent control points remains unchanged, and $d_{i}=∥{\mathrm{cp}}_{i}-{\mathrm{cp}}_{i+1}∥$ is calculated between the control points. In fact, the first term of the smoothing function treats all control points as a deformable elastic band and behaves as an internal contractive force to make the trajectory as evenly distributed on the straight line as possible. The second and third terms smooth the whole trajectory by minimizing higher-order derivatives.
(2) *Collision costs:* Since the initial trajectory may be close to the obstacles, the collision penalty function can keep the control point away from the obstacles by the repulsive action [7]. Therefore, the collision penalty term is defined as
  (15) $$C_{c}=\sum_{i=k}^{N-k}VF\left(f\left(d({\mathrm{cp}}_{i})\right)\right)$$
  (16) $$VF\left(f\left(d({\mathrm{cp}}_{i})\right)\right)={\left(f\left(d({\mathrm{cp}}_{i})\right)-f_{\varepsilon }\right)}^{2}$$
  (17) $$f\left(d({\mathrm{cp}}_{i})\right)=\frac{k_{d}}{1+e^{\left(d({\mathrm{cp}}_{i})\left(2/d_{0}\right)-1\right)\alpha }}$$
  where $d({\mathrm{cp}}_{i})$ is the minimum Euclidean distance between control points and the obstacles, $f_{\varepsilon }$ is the repulsive force at the safe distance threshold $d_{0}$, $k_{d}$ represents the maximum magnitude of the repulsive force, and α(α≫1) is a shape parameter. This design allows the repulsive force magnitude $f\left(d({\mathrm{cp}}_{i})\right)$ to reach its maximum value $k_{d}$ when $d({\mathrm{cp}}_{i})=0$, while $f\left(d({\mathrm{cp}}_{i})\right)$ approaches 0 when $d({\mathrm{cp}}_{i})>d_{0}$ and no repulsive force is generated.
  To facilitate fast distance detection, the Euclidean distance field (EDF) of the occupancy volume is calculated by an efficient algorithm [48] with complexity O(n1), where $n1=N^{3}$ is the number of voxel grids, and *N* represents the size of the volume along a single axis. Furthermore, the trilinear interpolation technique is adopted to enhance the detection accuracy of distance [33], which compensates for voxel grid discretization errors and is beneficial for numerical optimization [41].
(3) *Feasibility costs:* The higher-order derivative of a B-spline curve is also a B-spline with the convex hull property. In other words, if the derivative control points are bounded within the convex hull, expanded by the maximum allowed derivative, then the derivative-spline is also bounded [43]. Based on this property, we ensure the feasibility of the trajectory by designing a penalty function that constrains the higher-order derivatives of the control points as follows:
  (18) $$C_{d}=\sum_{i=k-1}^{n-k}w_{v}G\left(v_{i}\right)+\sum_{i=k-2}^{n-k}w_{a}G\left(a_{i}\right)+\sum_{i=k-3}^{n-k}w_{j}G\left(j_{i}\right)$$
  where $w_{v}$, $w_{a}$, and $w_{j}$ are the weights of the penalty terms of velocity, acceleration, and jerk, respectively. The penalty term is defined as
  (19) $$G{\left(\beta \right)}_{\beta \in \{v,a,j\}}=\left\{\begin{array}{cc} \sum_{e\in \{x,y,z\}}{\left(\beta_{e}^{2}-\beta_{\max}^{2}\right)}^{2} & \beta_{e}^{2}>\beta_{\max}^{2}\\ 0 & \beta_{e}^{2}\leq \beta_{\max}^{2} \end{array}\right.$$

### 4.3. Numerical Optimization Method

The numerical solution of Equation (13) is obtained using the L-BFGS method [49], which uses curvature information to construct a Hessian approximation from the nearest iteration. Curvature information from earlier iterations that are not associated with the behavior of the current iteration Hessian is discarded to save storage. In the current work, the objective function is explicit and has separability, so the L-BFGS method that approximates the inverse Hessian from gradient information usually converges quickly and is robust [50]. Due to low computing requirements and small memory consumption, L-BFGS is suitable for real-time applications. The L-BFGS numerical optimization process is elaborated as follows:

Let the continuously differentiable unconstrained optimization problem be $\min_{x}f\left(x\right)$. The updating for x follows the approximate Newton steps [50]:

(20) $$x_{k+1}=x_{k}-\alpha_{k}H_{k}\nabla f_{k}$$

where $\alpha_{k}$ is the step length and satisfy the Wolfe condition, $H_{k}\nabla f_{k}$ is the search direction, and $H_{k}$ is updated at every iteration by means of the following formula:

(21) $$H_{k+1}=V_{k}^{T}H_{k}V_{k}+\rho_{k}s_{k}s_{k}^{T}$$

where $\rho_{k}=1/y_{k}^{T}s_{k},\;V_{k}=I-\rho_{k}y_{k}s_{k}^{T},\;s_{k}=x_{k+1}-x_{k}$ and $y_{k}=\nabla f_{k+1}-\nabla f_{k}$.

Furthermore, the inverse Hessian approximation is implicitly stored by storing a finite number of vector pairs $\left\{s_{i},y_{i}\right\}$ to avoid high storage and operation costs when the number of variables is large. The product of $H_{k}\nabla f_{k}$ is computed by a two-loop recursion updating algorithm [51], which performs an iterative inner product and vector sum operation over $\nabla f_{k}$ and the vector pair $\left\{s_{i},y_{i}\right\}$. In fact, only curvature information from the *m* (between 3 and 20) most recent iterations is included in the set of vector pairs.

The initial inverse Hessian $H_{k}^{0}$ for L-BFGS updating is chosen to follow [50], i.e.,

(22) $$H_{k}^{0}=\gamma_{k}I$$

where $\gamma_{k}=\frac{s_{k-1}^{T}y_{k-1}}{y_{k-1}^{T}y_{k-1}}$ is the scaling factor that attempts to estimate the size of the true Hessian matrix along the most recent search direction. Finally, the L-BFGS algorithm can be summarized and rewritten as Algorithm 2.
 **Algorithm 2** L-BFGS algorithm**Input:** Start point: $x_{0}$, the number of most recent iterations: *m*, k=0;**Output:** Optimal $x^{*}$  1:Initial $H_{k}^{0}$  2:**repeat**  3:    $p_{k}\leftarrow -H_{k}\nabla f_{k}$(by two-loop recursion updating algorithm)  4:    $x_{k+1}=x_{k}+\alpha_{k}p_{k}$ ($\alpha_{k}$ satisfy the Wolfe conditions)  5:    **if** k>m **then**  6:        Discard($\left\{s_{k-m},y_{k-m}\right\}$);  7:    **end if**  8:    $H_{k+1}=V_{k}^{T}H_{k}V_{k}+\rho_{k}s_{k}s_{k}^{T}$  9:    $\rho_{k}=1/y_{k}^{T}s_{k},\;V_{k}=I-\rho_{k}y_{k}s_{k}^{T},\;s_{k}=x_{k+1}-x_{k}$  10:    $s_{k}$← Computeandsave($x_{k+1},x_{k}$)  11:    $y_{k}$← Computeandsave($\nabla f_{k+1},\nabla f_{k}$)  12:     *k*←k+1  13:**until** ($f\left(x_{k+1}\right)>f\left(x_{k}\right)$)

### 4.4. Infeasible Derivative Control Points Adjustment Method

For a uniform B-spline, each knot span Δt is equal. Collision penalty tends to force larger separations between local control points close to obstacles. The robot needs to move farther in the same amount of time, which means the robot needs to move faster. Therefore, derivative control points that do not meet the feasibility requirements may appear. To this end, an adjustment method based on the derivative B-spline-bounded sufficient condition [43] and the convex hull property is designed, which locally adjusts the infeasible derivative control points to within the maximum allowable bound.

As in Equation (10), the position coordinate c(ξ) of B-spline curve in the *s*-th control point span can be expressed as:

(23) $$\begin{array}{c} c_{s}\left(\xi \right)=b^{⊤}M_{k}Y_{s} \end{array}$$

Let the mapping matrix be $C_{ld}$, which satisfies $\frac{db^{⊤}}{d^{ld}\xi }=b^{⊤}{\left(C_{ld}\right)}^{⊤}$; then, the corresponding derivative control points can be derived following [32]:

(24) $$\frac{dc_{s}\left(\xi \right)}{d^{ld}\xi }=\frac{1}{{\left(\nabla t\right)}^{ld}}\frac{db^{T}}{d^{ld}\xi }M_{k}Y_{s}=\frac{1}{{\left(\nabla t\right)}^{ld}}b^{T}C_{ld}^{T}M_{k}Y_{s}$$

Let $S_{ld}=M_{k}^{-1}C_{ld}M_{k}/{\left(\Delta_{t}\right)}^{ld}$, then we have

(25) $$\frac{dc_{s}\left(\xi \right)}{d^{ld}\xi }=b^{⊤}M_{k}\left(S_{ld}Y_{s}\right)$$

It can be seen from Equation (25) that the derivative curve is also a B-spline curve with $S_{ld}Y_{s}$ as the control point span. If $\left|S_{ld}Y_{s}\right|\leq \lambda_{ld}^{\max}1_{k\times 1}$, then the derivative control points are completely contained in the range from $-\lambda_{ld}$ to $\lambda_{ld}$. Using the above derivation and the B-spline curve convex hull property, an infeasible derivative control point adjustment method is designed by adjusting the infeasible derivative control points to be within the convex hull of the maximum allowable derivative expansion. An optional adjustment scaling factor η is set as follows:

(26) $$\eta_{r}=\frac{sgn\left(r_{\mu }^{\inf}\right)r_{\mu }^{\max}}{r_{\mu }^{\inf}},\;\mu \in \left\{x,y,z\right\},r\in \left\{v,a,j\right\}$$

where $sgn\left(r_{\mu }^{\inf}\right)$ indicates the symbol of the infeasible derivative control point, $r_{\mu }^{\max}$ is the maximum allowable value, and $r_{\mu }^{\inf}$ is the value of the infeasible control point. Taking the adjustment of a infeasible velocity control point as an example,

(27) $$v_{\mu }^{\max}=\eta_{v}v_{\mu }^{\inf}=\frac{sgn\left(v_{\mu }^{\inf}\right)v_{\mu }^{\max}}{v_{\mu }^{\inf}}v_{\mu }^{\inf}\in \left[-v_{\mu }^{\max},v_{\mu }^{\max}\right]$$

The adjustment of infeasible acceleration and jerk follows a similar process as well. Finally, infeasible derivative control points can become feasible by scaling factor adjustment. Then, the whole derivative spline is feasible according to the B-spline convex hull property.

### 4.5. Local Replanning Strategy

**Figure 3:**
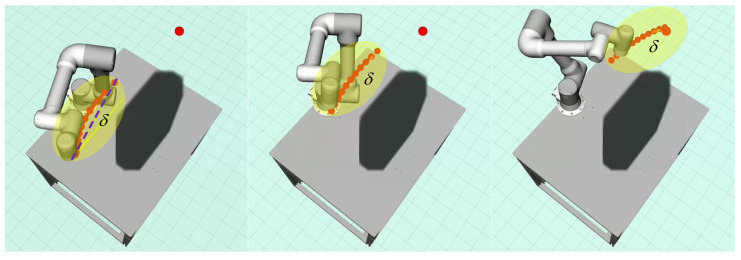
The receding-horizon-based replanning strategy generates a trajectory from the starting point to the goal, segmented by a preset searching radius δ.

In an open industrial cell, it may be inefficient to directly generate the global trajectory from the starting point to the goal, since the part of the trajectory occupied by dynamic obstacles is never executed. To improve efficiency, following [41,43], a replanning strategy using receding horizon framework is adopted to generate a trajectory from the start point to the goal segmentally by a predefined searching radius δ. In other words, the path search is only performed in a spherical region centered on the current position of the end-effector and with a radius δ. An illustrative example is shown in Figure 3. Once the motion primitive node exceeds the search radius, the search process will be stopped, then the trajectory optimization and infeasible derivative control points adjustment will be performed.

The replanning process is activated in both active and passive modes. In the active mode, the replanner is invoked at a regular interval and updates the trajectory with the latest environmental information. In passive mode, the replanner is activated by collision detection, i.e., once the current planned trajectory collides with obstacles, the replanner will be triggered to ensure that a new safe trajectory is available.

## 5. Collision Avoidance Optimization for the Robot Links

**Figure 4:**
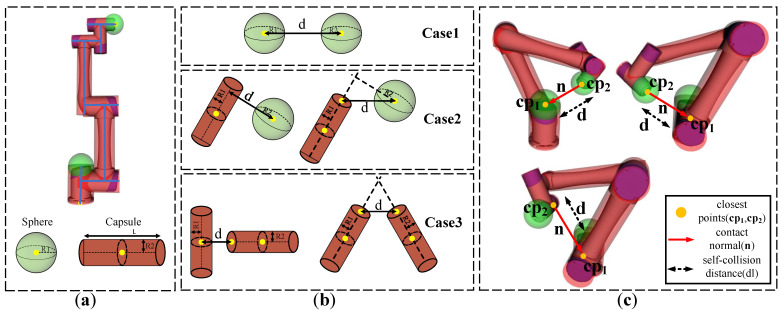
Illustration of robot self-collision avoidance. (**a**) The robot model is approximated as a simple 3D geometric model. (**b**) Geometric model distance evaluation cases. (**c**) Examples of self-collision and descriptions of the closest points ${\mathrm{cp}}_{1},{\mathrm{cp}}_{2}$ and contact normal n.

In this section, the collision avoidance problems between links and obstacles, and the robot itself, are discussed, respectively. The former avoids the collisions between the robot links and obstacles, and the latter avoids the robot from self-collision during motion. Collision detection needs to be performed first. To reduce CPU consumption, the robot is approximated as a simple 3D sphere and cylinder as shown in Figure 4a. The sphere is characterized by the center and radius, R1. The cylinder is characterized by the center, axis length, *L*, and radius, R2. For self-collision avoidance, collision detection can be divided into three cases, as shown in Figure 4b:

- Collision detection between spheres (Case 1): The distance, *d*, between the centers of two spheres is measured to detect whether the two spheres collide. If $d>\left(R1+R2\right)$, where R1 and R2 are the radii of the two spheres, then the two spheres do not collide.
- Collision detection between a cylinder and a sphere (Case 2): The center of the sphere is projected onto the axis of the cylinder to detect collisions between the cylinder and the sphere. Two cases need to be discussed. First, if the projection of the sphere center is inside the cylinder, the distance, *d*, between the sphere center and its projection on the cylinder axis is considered as the collision detection distance. If $d>\left(R1+R2\right)$, the cylinder and the sphere do not collide. Second, if the projection of the sphere center is outside the cylinder, the distance, *d*, between the sphere center and the nearest cylinder end will be used as collision detection distance. If $d>\left(R1+R2\right)$, then no collision occurs.
- Collision detection between cylinders (Case 3): The cylinders are reduced to two axes to detect collisions between two cylinders. Again, two possible cases that need to be discussed. First, if the intersection of the two cylinders axes is inside the first cylinder, the distance, *d*, between the closest points is used as the collision detection distance. If $d>\left(R1+R2\right)$, the two cylinders do not collide. Second, if the intersection of two cylinders axes is outside the cylinders, then the closest distance is determined by the distance, *d*, between the ends of the two cylinders. If $d>\left(R1+R2\right)$, then no collision occurs.

To avoid robot self-collision, the distances between the links that may collide need to be calculated. Denote the Cartesian space positions of the simplified geometric axes at configuration $q\in R^{n}$ as CS(q), and the Euclidean distance vector from $P_{i}$ to $P_{j}$ as $d\left(P_{i},P_{j}\right):R^{3}\times R^{3}\to R^{3}$, then the self-collision distance, dl, between robot components that may collide, can be defined as

(28) $$\begin{array}{cc} dl=\min & ∥d\left(P_{i},P_{j}\right)∥\\ s.t.\;\; & P_{i},P_{j}\in CS\left(q\right),\;i,j\in \left\{Link_{1},Link_{2},\cdots ,Link_{6}\right\} \end{array}$$

where the position of the cylinder axis and the position of the sphere center can be easily obtained by the forward kinematics model of the robot. Due to the structural constraints of the robot itself, some link components never collide (including adjacent links). Therefore, we divide the link collision states into two categories: never-in-collision state, N, and possible-collision state, M. To speed up self-collision detection, only components that may collide with other components are detected. Pairs of link components that require self-collision detection are listed as shown in Table 2.

Since the robot motion interpolation is discretized, we need to ensure that the robot’s next movement distance is less than the minimum self-collision detection distance, dl, to avoid self-collision. The Cartesian velocity vector along the contact normal n is calculated to predict the distance the robot will move next. The Cartesian velocity vector is calculated by

(29) $$x_{chain}=J_{chain}\dot{q}$$

where $J_{chain}$ is the Jacobian of the closest point closer to the end-effector. The main reason is that self-collisions are usually caused by end-effector manipulation tasks. Figure 4c shows the closest points ${\mathrm{cp}}_{1}$ and ${\mathrm{cp}}_{2}$, where ${\mathrm{cp}}_{2}$ is closer to the end-effector.

Once the self-collision distance, dl, is detected and the closest point Cartesian velocity $x_{chain}$ is obtained, then $x_{chain}$ is projected onto the contact normal n and multiplied by the time step Δt; the self-collision avoidance constraint is defined as

(30) $$\Delta t*n^{T}\left(J_{chain}\dot{q}\right)<\left(dl-d_{l0}\right)$$

where the contact normal n is the direction vector of the distance vector between the two closest points. $d_{l0}$ is the safe distance threshold of the self-collision. The above formula shows that the distance of the closest point moving along the contact normal direction in time step Δt is less than the self-collision distance. Several illustrative examples of self-collision avoidance are also shown in Figure 4c, which correspond to the three collision detection cases shown in Figure 4b.

**Figure 5:**
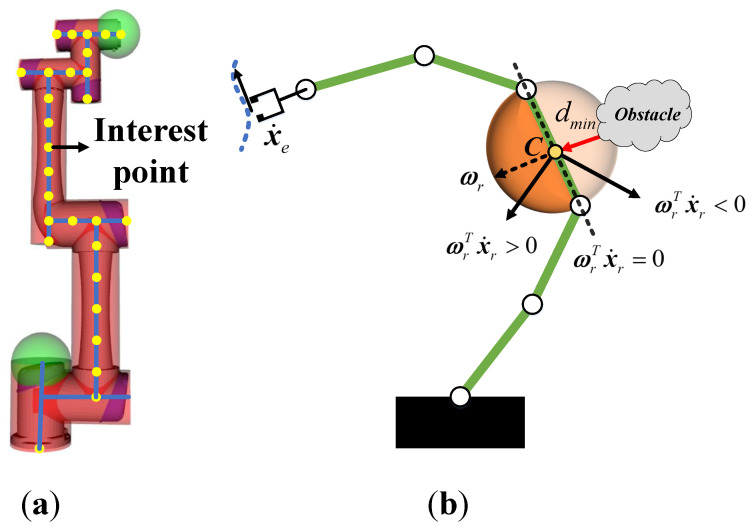
(**a**) The interest points are the yellow points along with the links of the robot and the envelope spheres and cylinders constitute the safety protection zone. (**b**) A safe feasible set example of the robot links collision avoidance.

For the links collision avoidance, as with the self-collision method, spheres and cylinders surrounding the robot body are used to provide safety contours. As shown in Figure 5a, the interest points (yellow) distributed along the links are set for collision detection between the links and obstacles. Different from the traditional collision avoidance method based on the concept of artificial potential field, it only makes the robot avoid obstacles along the distance vector direction. In the current work, a constraint-relaxed links collision avoidance method is adopted by solving a standard quadratic programming problem, which minimizes the deviation between the actual trajectory and the back-end optimized trajectory under the constraints of links collision avoidance and self-collision avoidance. As shown in Figure 5b, the hemispherical area (dark orange) centered on the interest point C is the feasible set of collision avoidance.

Specifically, the center $\omega_{r}$ of the feasible set of single interest point can be configured as:

(31) $$\omega_{r}=\frac{d}{∥d∥}$$

where d represents the minimum distance vector between the interest point and obstacles. To keep the interest point C away from obstacles, the component of collision avoidance velocity ${\dot{x}}_{r}$ of the interest point C along the vector $\omega_{r}$ needs to be non-negative, that is

(32) $${\omega_{ra}}^{T}{\dot{x}}_{r}={\omega_{ra}}^{T}J_{c}\dot{q}=J_{ro}\dot{q}\geq 0$$

where $\omega_{ra}\in R^{6\times 1}$ is the zero-filled augmented matrices of $\omega_{r}$. $J_{c}$ is the Jacobian of the interest point C. $J_{ro}$ indicates the Jacobian matrix after dimensionality reduction.

Since there may be an infinite number of collision avoidance velocities satisfying Equation (32) in the feasible set space, the optimal collision avoidance velocity command cannot be manually selected. In addition, the self-collision avoidance constraint Equation (30) also needs to be satisfied during the links collision avoidance process. To solve the above problems, we integrate multiple tasks into quadratic programming (QP) framework with inequality constraints, as follows:

(33) $$\begin{array}{cc} \; & \min_{\dot{q}}\;{∥{\dot{x}}_{op}-J\dot{q}∥}_{2}^{2}\\ \; & \;s.t.\;\;J_{ro}\dot{q}\geq 0\\ \; & \;\;\;\;\;\;\;\;-\;\left(nJ_{chain}\right)\dot{q}+\frac{dl-d_{l0}}{\Delta t}>0\\ \; & \;\;\;\;\;\;\;\;{\dot{q}}_{\min}\leq \dot{q}\leq {\dot{q}}_{\max} \end{array}$$

where the objective function minimizes the velocity deviation of the end-effector from actual to desired under the constraints. ${\dot{x}}_{op}$ is the desired velocity profile generated by the back-end optimization step, $J\dot{q}$ is the end-effector actual velocity profile. ${\dot{q}}_{\min}$ and ${\dot{q}}_{\max}$ are the minimum and maximum allowable joint velocities, respectively. Equation (33) is a standard quadratic programming problem, and its Hessian matrix $H=J^{T}J$ is symmetric positive definite, so there is a global optimal solution. The optimal solution is composed of collision avoidance joint velocity and compensation joint velocity. The velocities of the joints before the constrained interest point are used to avoid the robot from colliding with obstacles, and the velocities of the following joints are used as compensation values to track the desired velocity profile.

It is worth noting that Equation (33) involves the back-end trajectory optimization step and the links collision avoidance step, where the optimized task space trajectory is used as the desired trajectory. More specifically, if the links are not constrained by obstacles or have no risk of self-collision, the robot will normally execute the optimized task space trajectory. Instead, the link collision avoidance optimization will be activated to achieve link collision avoidance while tracking the optimized task space trajectory.

## 6. Simulation and Real-World Experiment Results

In this section, we first verify the effectiveness of the proposed algorithm in a variety of different simulation scenarios, which include static obstacles as well as dynamic obstacles. We benchmarked the proposed algorithm against existing state-of-the-art motion planners and trajectory optimization algorithms. We first compare our method with two state-of-the-art motion planners for manipulator arms, RRT*^X^* [27] and the DRGBT [30]. Second, we compare our method with two state-of-the-art trajectory optimization algorithms, CHOMP [16] and TrajOpt [17], which are popular in the field of industrial robot trajectory optimization and integrated into the open-source motion planning framework MoveIt [52]. We choose these benchmark methods due to their superior performance, reproducibility, and code availability. Compared with the advanced motion planners RRT*^X^* and DRGBT, our method can generate a shorter and smoother path in shorter time with a higher success rate. Compared with the advanced trajectory optimization algorithms CHOMP and TrajOpt, our method can generate a smoother optimization trajectory with a higher success rate and is more suitable for real-time applications. Finally, we also demonstrate the effectiveness of our method on a real-world robotic pick-place task. More experimental details are also presented in the Supplementary Materials Video S1, and the download link is in the Supplementary Materials section of the paper.

### 6.1. Experimental Settings

**Figure 6:**
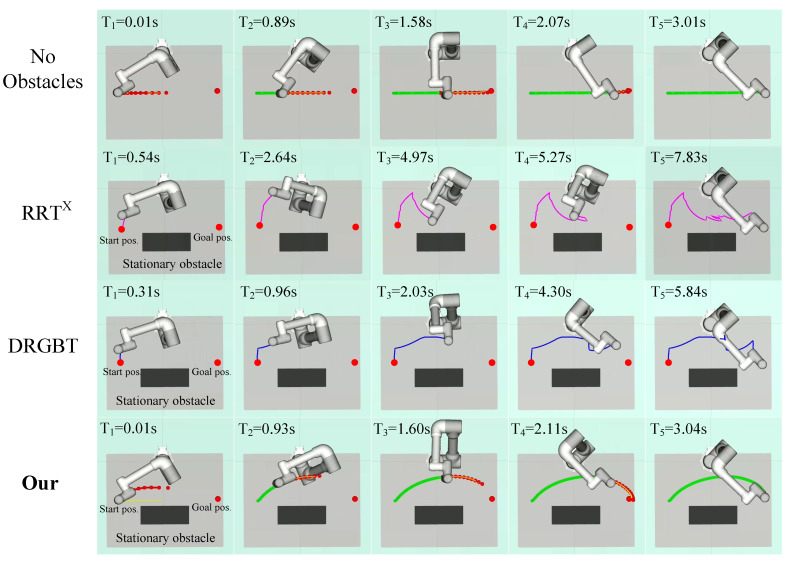
Visualization of robot motion trajectories in a single static obstacle comparison experiment. The first row: the original motion trajectory of our method without obstacles. The second row: the motion trajectory of the RRT*^X^* algorithm. The third row: the motion trajectory of DRGBT algorithm. The fourth row: the motion trajectory of our algorithm.

The experiments were performed on a 6-DOF collaborative robot, the model of which is shown in Figure 6. The size of the robot workspace is 3×3×3 m, which is modeled as a 3D grid map with a resolution of 1 mm. Too large a resolution may increase the discrete error, and too small a grid resolution may increase the path search time. Therefore, the choice of grid resolution is a compromise between discrete error and path search time. The choice of grid resolution in the current work fully considers the two factors. The start and goal positions in Cartesian space are $p_{s}=\left(0.3406,-0.3647,0.4318\right)$ and $p_{g}=\left(0.3289,0.4763,0.5000\right)$, respectively, which are predefined. The Euclidean distance is used as a safety metric. The safe distance threshold between the robot and the obstacles is $d_{0}=0.14$ m, and the self-collision safe distance threshold is $d_{s}=0.05$ m. A greater safety distance will cause the robot to move more conservatively but may increase the task execution time and trajectory length, thereby reducing work efficiency. Therefore, the setting of the safety distance is a compromise between safety and efficiency.

The weights of the trajectory optimization cost function are set to $\varphi_{s}=8$, $\varphi_{c}=0.3$, and $\varphi_{d}=0.01$. The weight of each optimization term indicates its relative importance. The selection of weights is a compromise between the cost of each optimization item to minimize the total optimization cost. The proposed algorithm already considers the safety and dynamic feasibility of the initial trajectory in the kinodynamic path search step, while the trajectory smoothness is not considered. Therefore, the smoothing cost is applied with greater weight in the trajectory optimization objective function, while the costs of safety and dynamic feasibility are given smaller weights, respectively. Similarly, we apply a greater weight to the jerk penalty term in the feasibility penalty function and smaller weights to the penalties of the velocity and acceleration, respectively. The weights of velocity, acceleration, and jerk in the feasibility penalty function are set as $\omega_{v}=0.01$, $\omega_{a}=0.01$ and $\omega_{j}=0.1$. The repulsive force magnitude in the collision penalty function is $k_{d}=0.1$, α=6. A cubic B-spline curve is used in the back-end trajectory optimization step, i.e., k=3.

All simulations are performed on a laptop with Intel Core i7-9750 CPU @ 2.6 GHz and 8 GB memory running Ubuntu 18.04 and ROS Melodic, and the programming language uses C++. The real-world experiments are carried out on a physical 6-DOF collaborative robot. The robotic workspace is surrounded by two Kinect cameras to detect workspace obstacles. The experimental parameter settings are the same as the above simulation settings. The online trajectory generation algorithm is deployed on an external PC, and the PC and the robot controller communicate through a 1 kHz network port.

### 6.2. Simulation Experiments

#### 6.2.1. Scenes with a Single Static and Dynamic Obstacle

**Figure 7:**
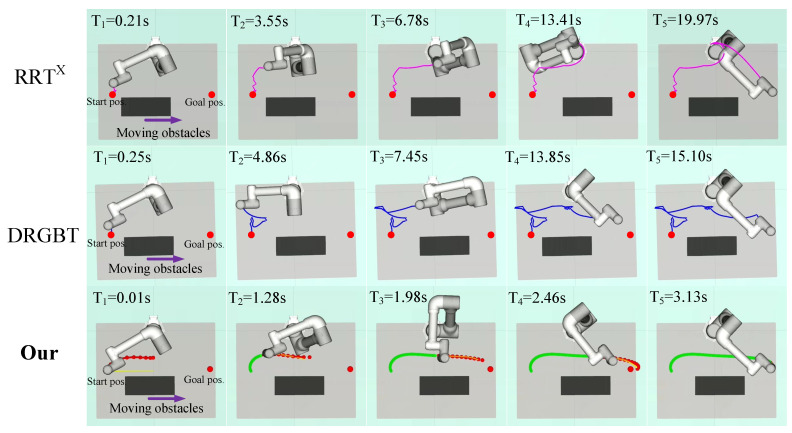
Visualization of robot motion trajectories in a single dynamic obstacle comparison experiment. The first row: the motion trajectory of the RRT*^X^* algorithm. The second row: the motion trajectory of the DRGBT algorithm. The third row: the motion trajectory of our algorithm.

In the first scenario, there is only one static obstacle in the workspace, but it is so close to the robot that the original trajectory may become infeasible (see Figure 6). The robot wants to run from the starting position to the goal position safely and efficiently, the planned trajectory should not only be collision-free to ensure safety but also shorten the planning time as much as possible to improve the robot’s work efficiency. In the second scenario, a single obstacle reciprocates between the start and goal positions at a speed of 0.03 m/s, periodically blocking the robot’s motion. At this time, the trajectory generation algorithm should ensure that the search path is collision-free and meets the real-time requirements. Figure 6 and Figure 7 qualitatively show the experimental results of our method and the benchmark motion planners in a single static and dynamic obstacle scene, respectively.

From the qualitative experimental results in Figure 6, it can be seen that there are some sudden turning points in the planned paths of both the RRT*^X^* algorithm and the DRGBT algorithm, and the overall trajectories are not smooth, while the trajectory generated by our method is smoother and shorter in length. In terms of total runtime, our method only takes about 3.04 s, while the RRT*^X^* algorithm and the DRGBT algorithm take about 7.83 s and 5.84 s, respectively. Furthermore, as can be seen from the qualitative experimental results of a single dynamic obstacle shown in Figure 7, although both our method and the benchmark motion planners can search for collision-free paths, our method shows superiority and robustness. From the overall path of the search, the search path of our method is still smooth and short, while the search paths of the RRT*^X^* algorithm and DRGBT algorithm are tortuous and have large fluctuations. From the total runtime, our method takes only about 3.13 s, almost the same as the static scenario, which shows the robustness of our method. The total running times of the RRT*^X^* algorithm and the DRGBT algorithm are 13.97 s and 15.10 s, respectively, which are much larger than the time taken by our method.

#### 6.2.2. Scenes with Multiple Static and Dynamic Obstacles

**Figure 8:**
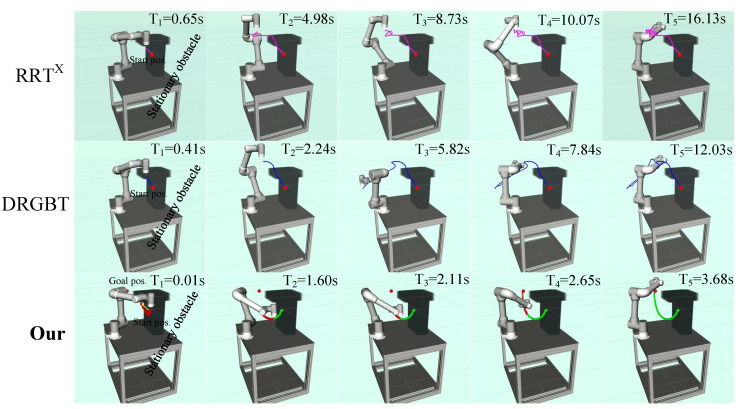
Visualization of robot motion trajectories in a multiple static obstacles comparison experiment. The first row: the motion trajectory of the RRT*^X^* algorithm. The second row: the motion trajectory of the DRGBT algorithm. The third row: the motion trajectory of our algorithm.

**Figure 9:**
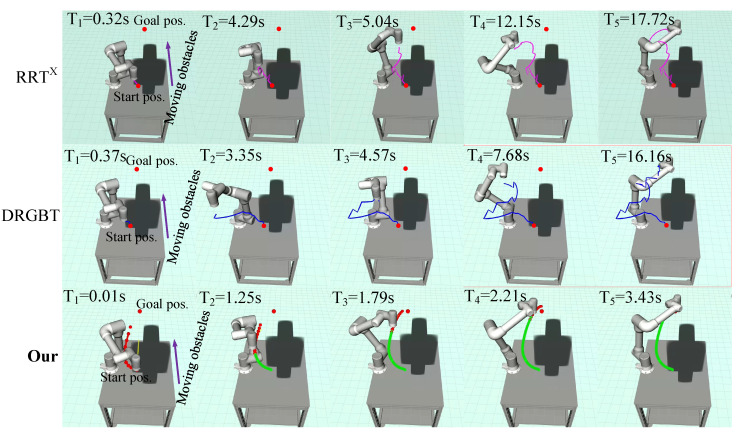
Visualization of robot motion trajectories in a multiple dynamic obstacles comparison experiment. The first row: the motion trajectory of the RRT*^X^* algorithm. The second row: the motion trajectory of the DRGBT algorithm. The third row: the motion trajectory of our algorithm.

In the third case, two static obstacles parallel and perpendicular to the tabletop approach the robot. The start and goal positions are the same as in the first scene. Unlike the single static obstacle scenario, obstacles parallel to the tabletop impose constraints on the robot trajectory, reducing the robot’s feasible space. In the fourth scenario, two dynamic obstacles perpendicular to each other and close to the robot reciprocate at a speed of 0.03 m/s between the starting position and the goal position, which imposes constraints on the motion trajectory in a more complex form. It is a challenging task as the robot needs to replan the motion trajectory in real time to quickly bypass obstacles in both vertical sections and then converge to the goal configuration. Figure 8 and Figure 9 qualitatively show the experimental results of our method and the benchmark motion planners in scenes with multiple static and dynamic obstacles, respectively.

Figure 8 shows the path search results of our method and the benchmark motion planners in a scene with two static obstacles. It can be seen that our method outperforms the RRT*^X^* algorithm and the DRGBT algorithm in both total path length and total runtime. In terms of path length, our method generates a trajectory that bypasses obstacles from below the obstacle that parallels to the tabletop, greatly shortening the total running path length, in line with human intuition. Compared with our method, the trajectories generated by the RRT*^X^* algorithm and the DRGBT algorithm seem to have unnecessary deflections so that the total path length may be larger. In terms of total runtime, the total runtime of our method is significantly smaller than that of the RRT*^X^* algorithm and that of the DRGBT algorithm, with little change compared to the previous test scene time, which further demonstrates the robustness of our method. Figure 9 shows the path search results of our method and the benchmark motion planners in a scene with two dynamic obstacles. It can be seen that our method is superior to the RRT*^X^* algorithm and the DRGBT algorithm in both search path quality and total runtime. In terms of path quality, our method does not exhibit abrupt turns due to multiple dynamic obstacle constraints but stably converges to the goal. In terms of total runtime, our method takes only 3.43 s from the starting point to the goal point, while the RRT*^X^* algorithm and the DRGBT algorithm take about 11.81 s and 16.16 s, respectively. The total running time of both benchmark planners are more than three times that of our method.

#### 6.2.3. Quantitative Evaluation and Analysis of Simulation Results

The path search algorithms are also quantitatively compared in terms of algorithm success rate, single iteration time, total runtime, and Cartesian space path length. Each algorithm was run 100 times in each scenario to precisely obtain each evaluation index. The RRT*^X^* algorithm is an asymptotically optimal single-query replanning algorithm that refines and repairs the same search graph using the obstacles or robot change information. The existing search graph is quickly reconstructed through a graph rewiring cascade to repair its shortest-path subtree to the target. Therefore, RRT*^X^* needs to continuously update and repair the global search graph according to changes in the environment or robot position. The DRGBT algorithm is based on an adaptive horizon setting through predefined C-space path target nodes, where each node is assigned a weight determined by relative distance and captured environmental changes. This setting requires the algorithm to perform constant distance queries to modify local paths. In fact, both the RRT*^X^* algorithm and the DRGBT algorithm are essentially sampling-based path-planning methods, which obtain geometric path information without including time information. However, due to limited sampling, the quality of the planned path is not ideal. We also observed some unpredictable stochastic behaviors, as shown by large fluctuations and redundancy in the planned trajectories in Figure 7, Figure 8 and Figure 9. Since the path search costs of benchmark planners are evaluated without considering the control cost, the search paths may not be smooth. Our method takes into account the nonstatic initial state of the robot by integrating the control input over the duration to obtain the position, velocity, and acceleration state of each node. Since the search cost is evaluated by minimizing the control and time costs, the search path is time-minimized and exhibits good smoothness.

Table 3 quantitatively shows the experimental results of each evaluation index in the four research scenarios. In terms of success rate, both the proposed method and the DRGBT algorithm can successfully reach the target point in each run in the first and third scenarios, while the success rate of our approach is higher than that of the DRGBT algorithm in the second and fourth scenarios. The RRT*^X^* algorithm can successfully reach the target point in each run in the first scenario, while the success rate in other scenarios is lower compared to our method and the DRGBT algorithm. In terms of single iteration time, we show the maximum time, average time, and runtime standard deviation of the proposed method and the benchmark motion planners in four scenarios, respectively. From the experimental results, the single iteration time of our method significantly outperforms the benchmark motion planners. This shows that our method can quickly adjust the local trajectories in a very short time, which also explains why our method has a higher success rate than the benchmark motion planners in the second, third, and fourth scenarios. The randomized behavior of the sampling-based planner may also be another factor for the low success rate of the RRT*^X^* and DRGBT algorithms in dynamic environments. In terms of total runtime, the proposed method is also smaller than the benchmark motion planners, which means that our method can reach the target point from the starting point faster. In terms of path lengths in Cartesian space, the search path of our method is significantly smaller than those of the benchmark motion planners. The main reason is that the randomized behavior of the RRT*^X^* algorithm and the DRGBT algorithm may result in unpredictable performance, especially with a limited number of samples.

### 6.3. Comparison of Trajectory Optimization

**Figure 10:**
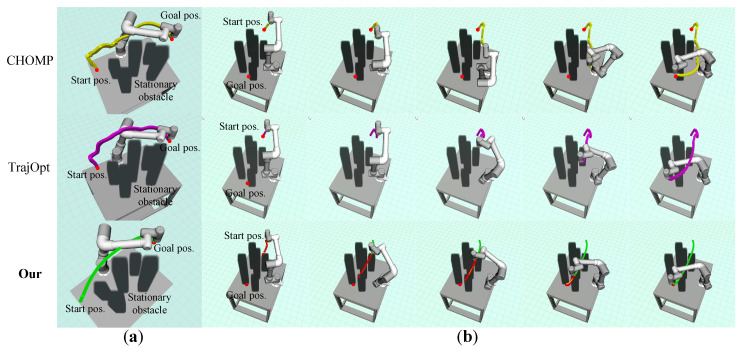
The visualization of the robot motion trajectories in the trajectory optimization comparison experiment. The first row: the motion trajectory of the CHOMP algorithm. The second row: the motion trajectory of the TrajOpt algorithm. The third row: the motion trajectory of our method.

For trajectory optimization, we benchmarked our method against existing state-of-the-art trajectory optimization algorithms, the CHOMP algorithm [16], and the TrajOpt algorithm [17], which are widely used for manipulator trajectory optimization. We created a simulated environment consisting of multiple staggered static obstacles, and the robot needs to move from a starting position to a goal position safely and efficiently. For fairness, our method and the benchmark optimization algorithms are validated in the same experimental environment. Figure 10 qualitatively shows the experimental results of our method and the benchmark optimization algorithms. Figure 10a shows the whole trajectory executed by the robot. It can be seen that the motion trajectory generated by our method is smoother, and the overall trajectory length is shorter. Figure 10b also shows the screenshots of the robot performing the complete experiment.

We quantitatively compare our method with the benchmark optimization algorithms in terms of success rate, single computation time, and trajectory smoothness. We perform 300 experiments for each algorithm, and the average value of each evaluation index was counted as shown in Table 4. We observe that our method can obtain collision-free trajectories from the starting point to the target point in all experiments, while the CHOMP algorithm and TrajOpt algorithm have a failure rate of 27% and 17%, respectively. It is mainly because CHOMP directly generates the initial trajectory from the starting point to the target point without considering obstacles. Then, the CHOMP algorithm iteratively adjusts the initial trajectory, but the gradient descent may fall into the local minimum of the cost function. The TrajOpt algorithm represents a trajectory in discrete-time form, may require post-optimization for execution, and may not stay collision-free. Our method adopts a replanning strategy that optimizes only one segment of the trajectory at each time step to avoid local minima. In addition, the initial trajectory generated by our path search module is inherently collision-free, which further improves the success rate of the optimization algorithm. In terms of single computation time, our method only takes about 0.216 ms, while the CHOMP and TrajOpt algorithms require 56.7 ms and 69.2 ms, respectively. The main reason is the CHOMP algorithm requires multiple iterations to optimize the entire trajectory and the TrajOpt algorithm needs to evaluate the fine discretization costs of the trajectory. The proposed method optimizes the replanned trajectory only within the search horizon instead of optimizing all trajectory points, thus saving optimization time. The global trajectory may become infeasible due to the uncertainty in the motions of the dynamic obstacles, so optimizing the global trajectory may be ineffective. In terms of total optimization time, our method only takes about 0.0635 s, while the CHOMP and TrajOpt algorithms take about 5.574 s and 1.94 s, respectively—much more than the optimization time of our method. Although the single iteration time of CHOMP is shorter than that of TrajOpt, the total optimization time is longer, which may be due to the higher number of iterations than TrajOpt. Then, combined with the trajectory smoothness information, it can be found that our method can generate a smoother trajectory even with less optimization time.

### 6.4. Real-World Experiments

**Figure 11:**
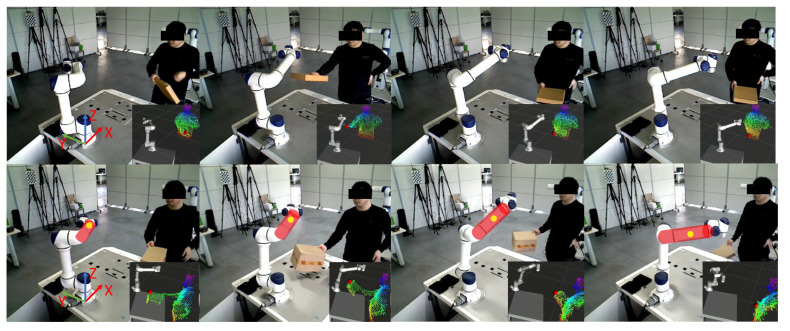
Real world experiments. The first and second lines show the experimental results of the end-effector and links collision avoidance, respectively.

**Figure 12:**
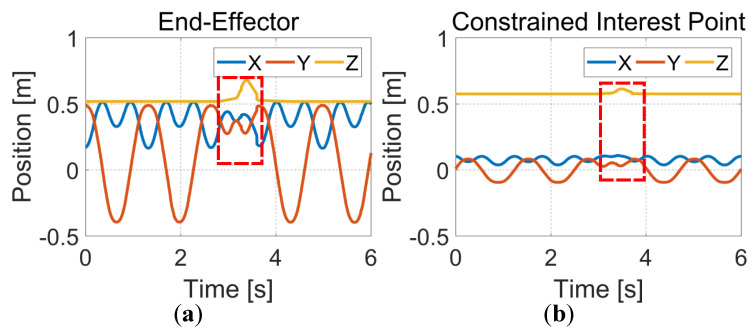
The visualization of the end-effector and constrained interest point motion trajectories. (**a**) The motion trajectory of end-effector. (**b**) The motion trajectory of the constrained interest point.

In this section, we validate the proposed method in the real world. A human partner holding an obstacle gradually approaches the moving robot in the current experimental setup. Figure 11 qualitatively shows the experimental results of online collision avoidance for physical robots. The first and second rows show the experimental results of the end-effector and links adjusting their trajectories online to avoid collision with obstacles, respectively. Figure 12 also quantitatively shows the trajectory changes of the end effector and a constrained interest point in the X, Y, and Z directions. From Figure 12a,b, it can be seen that the motion trajectories of the end effector and the constrained interest point are effectively adjusted and gradually converge to the target position (red dashed box).

**Figure 13:**
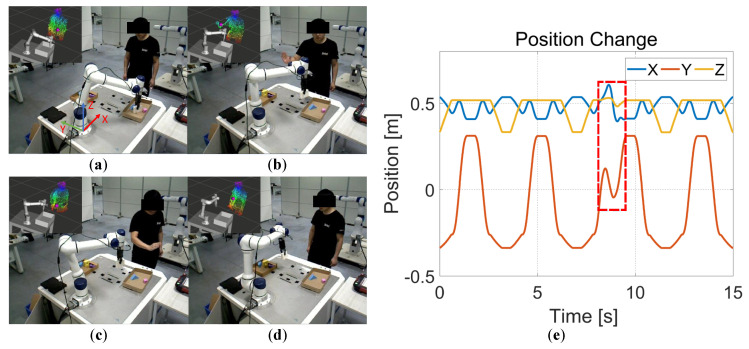
A pick-place experiment. (**a**) The robot starts to pick a item. (**b**) The human partner interferes with the robot. (**c**) The robot adjusts its motion trajectory to avoid obstacles. (**d**) The robot successfully avoids obstacles and transports the item to the placement point (red dotted box). (**e**) Trajectory change process of the end-effector.

In addition, we also deploy the proposed algorithm in a robot pick-place task. More specifically, when the robot picks up an item and places it in a placement box, the human partner interferes with the robot, and the robot need replan the motion trajectory to the placement box. Figure 13 shows a series of screenshots of the experimental process. Figure 13a indicates that the robot starts to pick an item; Figure 13b indicates that the human partner interferes with the robot when the robot is carrying the item; Figure 13c indicates that the robot adjusts its motion trajectory to avoid the human partner (red dotted box); and Figure 13d indicates that the robot successfully avoids the obstacle and transports the item to the placement box. Figure 13e also quantitatively shows the trajectory change process of the end-effector.

## 7. Conclusions

In this paper, we propose an efficient and complete online trajectory generation method to help a manipulator autonomous planning in dynamic environments. The overall framework is built bottom-up, and the trajectory generation problem is decoupled into front-end kinodynamic path search and back-end B-spline trajectory optimization modules. Given the current end-effector state, a series of discrete control inputs, and the links constraints, the front-end path search module generates a safe, smooth, and time-minimized initial trajectory. Then, a trajectory optimization method using the B-spline convex hull property is designed to increase the clearance between the trajectory and obstacles and improve the smoothness. To avoid the collision between the links and obstacles and the links themselves, the constraint-relaxed links collision avoidance method is integrated into the back-end optimization step by solving a standard quadratic programming problem. Finally, a complete real-time collision-free motion planning framework is developed to improve the safety and efficiency of robots working in unstructured dynamic environments.

## Figures and Tables

**Table 1:** A brief overview of related methods.

Methods		Source of Danger	Obstacle Representation	Real-Time	Convergence to Goal	Constraint Conditions (Safety, Smoothness, Dynamic Feasibility)
Potential field	[7,8]	Obstacle /human	Depth point	Y	N	Safety
	[9]	Obstacle /human	Point cloud	Y	Y	Safety
	[10]	Obstacle /human	Predefined position	Y	N	Safety
Danger field	[12,13,14]	Robot	Linear	Y	N	Safety
Safety field	[15]	Obstacle /human	Triangular mesh	Y	N	Safety
Optimization- based	[16]	Obstacle	3D voxel grids	N	Y	Safety, Smoothness
	[19]	Human	Human skeleton swept volumes	N	N	Safety
Learning- based	[20,23]	Obstacle	Predefined position	N	N	Safety
Replanning- based	[26]	Obstacle	Predefined position	Y (Additional GPU)	Y	Safety
	[27]	Obstacle	Predefined position	Y	Y	Safety
	[29,30]	Obstacle /human	Predefined position	Y	Y	Safety
	Ours	Obstacle /human	3D voxel grids	Y	Y	Safety, Smoothness, Dynamic feasibility

**Table 2:** Collision state relationship table of the links component pairs.

	Link 1	Link 2	Link 3	Link 4	Link 5	Link 6
Link 1	N	N	M	M	M	M
Link 2		N	N	M	M	M
Link 3			N	N	M	M
Link 4				N	N	N
Link 5					N	N
Link 6						N

**Table 3:** Quantitative results for four scenarios from the simulation study.

		Succ. Rate (%)	Single Iteration Time (s)			Traj. Time (s)			Path Length (m)		
			Mean	Max	Std	Mean	Max	Std	Mean	Max	Std
Scenario 1	RRT*^X^*	100%	0.179	0.287	0.0273	7.947	9.304	3.182	2.149	2.731	1.353
	DRGBT	100%	0.0117	0.162	0.0148	5.914	6.794	2.891	2.0126	2.516	1.263
	**Ours**	**100%**	**0.00562**	0.0104	0.000734	**3.076**	4.082	0.0849	**1.014**	1.204	0.483
Scenario 2	RRT*^X^*	65%	0.381	0.422	0.0329	20.074	23.634	4.551	2.749	3.338	1.775
	DRGBT	92%	0.1757	0.253	0.0218	16.237	20.525	4.272	2.6887	3.058	1.355
	**Ours**	**100%**	**0.00601**	0.0559	0.00887	**3.211**	4.116	0.103	**1.072**	1.211	0.479
Scenario 3	RRT*^X^*	87%	0.364	0.449	0.0299	15.886	17.376	3.313	2.713	3.134	1.544
	DRGBT	100%	0.2006	0.3662	0.0187	13.694	16.014	3.191	2.6065	3.724	1.346
	**Ours**	**100%**	**0.00573**	0.0113	0.00081	**3.128**	4.026	0.0957	**1.016**	1.207	0.425
Scenario 4	RRT*^X^*	21%	0.571	0.64	0.3011	18.633	23.912	5.047	3.184	4.267	1.774
	DRGBT	85%	0.3754	0.4563	0.2417	17.946	22.843	5.296	3.0795	4.096	1.536
	**Ours**	**94%**	**0.00639**	0.0715	0.0128	**3.371**	4.1299	0.141	**1.075**	1.259	0.498

**Table 4:** Quantitative evaluation results of the trajectory optimization comparison experiments.

	Succ. Rate (%)	Avg. Single Com. Time (s)	Mean Smoothness (m^2^/s^5^)	Total Optimization Time (s)
CHOMP	217/300	0.0567	158.91	5.574
TrajOpt	249/300	0.0692	97.67	1.942
**Ours**	300/300	0.000216	12.71	0.0635386

## Data Availability

Not applicable.

## Supplementary Materials

The following supporting information can be downloaded at https://www.mdpi.com/article/10.3390/e24050653/s1, Video S1: Simulation and real-world experiment demonstration videos.

## Nomenclature

κ	order of the derivative
$\omega_{r}$	center of the feasible set
A	state transition matrix
a	acceleration control point
B	input (control) matrix
b	basis vector
CP	control point span
${\mathrm{cp}}_{i}$	the *i*-th control point
C	close set of node
c(t)	B-spline curve
interestpoints	points of interest along the robot body
J	Jacobian matrix
j	jerk control point
$J_{c}$	Jacobian of a interest point
M	blending matrix
n	contact normal
P	open set of node
$p_{cur}$	current grid node
$p_{e}$	end-effector position in the task space
$p_{i}$	the *i*-th grid node
$q_{g}$	goal joints configuration
$q_{s}$	initial joints configuration
v	velocity control point
$x_{d}$	current state of the motion primitive
$x_{g}$	end-effector goal state
$x_{s}$	end-effector initial state
$Y_{s}$	the *s*-th control point span
$\chi_{li}$	geometric line segment representation of the *i*-th link
$\chi_{lk}$	geometric line segment representation of the *k*-th link
$\ddot{q}$	joint acceleration
Δu	discrete step of the control input
δ	replanning horizon size
$\dot{q}$	joint velocity
${\dot{x}}_{op}$	desired velocity profile generated by the back-end optimization step
${\dot{x}}_{r}$	repulsion velocity within feasible set
O	obstacle
μ	any axis in x,y,z
ρ	importance of the trajectory duration *T* relative to trajectory smoothness
τ	duration
φ	weight factor of the objective function
ξ	normalized knot span
$a_{r}$	coefficient of a polynomial trajectory
C(T)	search cost objective function
$C_{c}$	collision cost
$C_{d}$	feasibility cost
$C_{s}$	smoothness cost
$d({\mathrm{cp}}_{i})$	minimum distance between the *i*-th control point and obstacles
$d\left(\chi_{lk}\left(q_{a}\right),\chi_{li}\left(q_{a}\right)\right)$	minimum distance between the *k*-th link and the *i*-th link
$d\left(\chi_{lk}\left(q_{a}\right),O\right)$	minimum distance between the link segments and the obstacles
$d_{0}$	safe distance threshold between the links and obstacles
$d_{l0}$	safe distance threshold of the self-collision
dl	self-collision distance
$f(d\left({\mathrm{cp}}_{i}\right))$	magnitude of the repulsive force between the *i*-th control point and the closest obstacle
$f_{c}$	search cost from the current node to the goal node
G(β)	penalty function
$g_{c}$	search cost from the starting node to the current node
*k*	degree
*l*	discrete factor
ld	the ld-th derivative of a B-spline curve
*M*	total number of motion primitives
m+1	number of B-spline knots
min_dis	minimum distance between points of interest and obstacles
n+1	number of B-spline control points
*r*	degree of a polynomial trajectory
$s_{\mu }\left(t\right)$	polynomial trajectory along each axis ($\mu \in \left\{x,y,z\right\}$)
sig	collision signal
$u_{max}$	maximum value of control input
VF	repulsive force
*w*	weight factor of the feasibility penalty function
$p_{e}\left(t\right)$	end-effector trajectory in the task space
$U_{d}$	discretized control input set
$u_{d}$	discretized control input
U	control input set
$u\left(t\right)$	control input
$B_{i,k}$	B-spline basis function corresponding to the control point ${\mathrm{cp}}_{i}$

## References

[1] Krüger J., Lien T., Verl A. (2009). Cooperation of human and machines in assembly lines. CIRP Ann..

[2] Ajoudani A., Zanchettin A.M., Ivaldi S., Albu-Schäffer A., Kosuge K., Khatib O. (2018). Progress and prospects of the human–robot collaboration. Auton. Robot..

[3] Haddadin S., De Luca A., Albu-Schäffer A. (2017). Robot Collisions: A Survey on Detection, Isolation, and Identification. IEEE Trans. Robot..

[4] Villani V., Pini F., Leali F., Secchi C. (2018). Survey on human–robot collaboration in industrial settings: Safety, intuitive interfaces and applications. Mechatronics.

[5] Pairet È., Ardón P., Mistry M., Petillot Y. (2019). Learning generalizable coupling terms for obstacle avoidance via low-dimensional geometric descriptors. IEEE Robot. Autom. Lett..

[6] Li S., Han K., Li X., Zhang S., Xiong Y., Xie Z. (2021). Hybrid Trajectory Replanning-Based Dynamic Obstacle Avoidance for Physical Human-Robot Interaction. J. Intell. Robot. Syst..

[7] Flacco F., Kröger T., De Luca A., Khatib O. A depth space approach to human-robot collision avoidance. Proceedings of the 2012 IEEE International Conference on Robotics and Automation.

[8] Nascimento H., Mujica M., Benoussaad M. Collision Avoidance in Human-Robot Interaction Using Kinect Vision System Combined With Robot’s Model and Data. Proceedings of the 2020 IEEE/RSJ International Conference on Intelligent Robots and Systems (IROS).

[9] Tulbure A., Khatib O. Closing the loop: Real-time perception and control for robust collision avoidance with occluded obstacles. Proceedings of the 2020 IEEE/RSJ International Conference on Intelligent Robots and Systems (IROS).

[10] Lin H., Fan Y., Tang T., Tomizuka M. Human guidance programming on a 6-DoF robot with collision avoidance. Proceedings of the 2016 IEEE/RSJ International Conference on Intelligent Robots and Systems (IROS).

[11] Lin H.C., Liu C., Fan Y., Tomizuka M. Real-time collision avoidance algorithm on industrial manipulators. Proceedings of the 2017 IEEE Conference on Control Technology and Applications (CCTA).

[12] Lacevic B., Rocco P. Kinetostatic danger field—A novel safety assessment for human-robot interaction. Proceedings of the 2010 IEEE/RSJ International Conference on Intelligent Robots and Systems.

[13] Lacevic B., Rocco P., Zanchettin A.M. (2013). Safety Assessment and Control of Robotic Manipulators Using Danger Field. IEEE Trans. Robot..

[14] Zanchettin A.M., Lacevic B., Rocco P. A novel passivity-based control law for safe human-robot coexistence. Proceedings of the 2012 IEEE/RSJ International Conference on Intelligent Robots and Systems.

[15] Parigi Polverini M., Zanchettin A.M., Rocco P. Real-time collision avoidance in human-robot interaction based on kinetostatic safety field. Proceedings of the 2014 IEEE/RSJ International Conference on Intelligent Robots and Systems.

[16] Zucker M., Ratliff N., Dragan A.D., Pivtoraiko M., Klingensmith M., Dellin C.M., Bagnell J.A., Srinivasa S.S. (2013). CHOMP: Covariant Hamiltonian optimization for motion planning. Int. J. Robot. Res..

[17] Schulman J., Ho J., Lee A.X., Awwal I., Bradlow H., Abbeel P. Finding locally optimal, collision-free trajectories with sequential convex optimization. Proceedings of the Robotics: Science and Systems.

[18] Zanchettin A.M., Rocco P. (2017). Motion planning for robotic manipulators using robust constrained control. Control Eng. Practice.

[19] Ragaglia M., Zanchettin A.M., Rocco P. (2018). Trajectory generation algorithm for safe human-robot collaboration based on multiple depth sensor measurements. Mechatronics.

[20] Qureshi A.H., Simeonov A., Bency M.J., Yip M.C. Motion Planning Networks. Proceedings of the 2019 International Conference on Robotics and Automation (ICRA).

[21] Xu Z., Zhou X., Wu H., Li X., Li S. (2022). Motion Planning of Manipulators for Simultaneous Obstacle Avoidance and Target Tracking: An RNN Approach With Guaranteed Performance. IEEE Trans. Ind. Electron..

[22] Song Q., Li S., Bai Q., Yang J., Zhang A., Zhang X., Zhe L. (2021). Trajectory Planning of Robot Manipulator Based on RBF Neural Network. Entropy.

[23] Shen Y., Jia Q., Huang Z., Wang R., Fei J., Chen G. (2022). Reinforcement Learning-Based Reactive Obstacle Avoidance Method for Redundant Manipulators. Entropy.

[24] Liu H., Qu D., Xu F., Zou F., Song J., Jia K. A Human-Robot Collaboration Framework Based on Human Motion Prediction and Task Model in Virtual Environment. Proceedings of the 2019 IEEE 9th Annual International Conference on CYBER Technology in Automation, Control, and Intelligent Systems (CYBER).

[25] Hauser K. (2012). On responsiveness, safety, and completeness in real-time motion planning. Auton. Robot..

[26] Sun W., Patil S., Alterovitz R. (2015). High-frequency replanning under uncertainty using parallel sampling-based motion planning. IEEE Trans. Robot..

[27] Otte M., Frazzoli E. (2016). RRTX: Asymptotically optimal single-query sampling-based motion planning with quick replanning. Int. J. Robot. Res..

[28] Völz A., Graichen K. (2019). A Predictive Path-Following Controller for Continuous Replanning With Dynamic Roadmaps. IEEE Robot. Autom. Lett..

[29] Pupa A., Arrfou M., Andreoni G., Secchi C. (2021). A safety-aware kinodynamic architecture for human-robot collaboration. IEEE Robot. Autom. Lett..

[30] Covic N., Lacevic B., Osmankovic D. Path Planning for Robotic Manipulators in Dynamic Environments Using Distance Information. Proceedings of the 2021 IEEE/RSJ International Conference on Intelligent Robots and Systems (IROS).

[31] Liu S., Watterson M., Mohta K., Sun K., Bhattacharya S., Taylor C.J., Kumar V. (2017). Planning Dynamically Feasible Trajectories for Quadrotors Using Safe Flight Corridors in 3-D Complex Environments. IEEE Robot. Autom. Lett..

[32] Ding W., Gao W., Wang K., Shen S. Trajectory Replanning for Quadrotors Using Kinodynamic Search and Elastic Optimization. Proceedings of the 2018 IEEE International Conference on Robotics and Automation (ICRA).

[33] Usenko V., von Stumberg L., Pangercic A., Cremers D. Real-time trajectory replanning for MAVs using uniform B-splines and a 3D circular buffer. Proceedings of the 2017 IEEE/RSJ International Conference on Intelligent Robots and Systems (IROS).

[34] Zhou B., Gao F., Pan J., Shen S. Robust Real-time UAV Replanning Using Guided Gradient-based Optimization and Topological Paths. Proceedings of the 2020 IEEE International Conference on Robotics and Automation (ICRA).

[35] Zhou B., Pan J., Gao F., Shen S. (2021). RAPTOR: Robust and Perception-Aware Trajectory Replanning for Quadrotor Fast Flight. IEEE Trans. Robot..

[36] Kappler D., Meier F., Issac J., Mainprice J., Cifuentes C.G., Wüthrich M., Berenz V., Schaal S., Ratliff N., Bohg J. (2018). Real-time perception meets reactive motion generation. IEEE Robot. Autom. Lett..

[37] Meguenani A., Padois V., Silva J.D., Hoarau A., Bidaud P. (2016). Energy based control for safe human-robot physical interaction. 2016 International Symposium on Experimental Robotics, Proceedings of the International Symposium on Experimental Robotics, Tokyo, Japan, 3–6 October 2016.

[38] Han L., Gao F., Zhou B., Shen S. Fiesta: Fast incremental euclidean distance fields for online motion planning of aerial robots. Proceedings of the 2019 IEEE/RSJ International Conference on Intelligent Robots and Systems (IROS).

[39] Kant K., Zucker S.W. (1986). Toward efficient trajectory planning: The path-velocity decomposition. Int. J. Robot. Res..

[40] Liu S., Atanasov N., Mohta K., Kumar V. Search-based motion planning for quadrotors using linear quadratic minimum time control. Proceedings of the 2017 IEEE/RSJ international conference on intelligent robots and systems (IROS).

[41] Zhou B., Gao F., Wang L., Liu C., Shen S. (2019). Robust and efficient quadrotor trajectory generation for fast autonomous flight. IEEE Robot. Autom. Lett..

[42] Mueller M.W., Hehn M., D’Andrea R. (2015). A Computationally Efficient Motion Primitive for Quadrocopter Trajectory Generation. IEEE Trans. Robot..

[43] Ding W., Gao W., Wang K., Shen S. (2019). An Efficient B-Spline-Based Kinodynamic Replanning Framework for Quadrotors. IEEE Trans. Robot..

[44] Piegl L., Tiller W. (1996). The NURBS Book.

[45] de Boor C. (1972). On calculating with B-splines. J. Approx. Theory.

[46] Qin K. General matrix representations for B-splines. Proceedings of the Pacific Graphics’ 98, Sixth Pacific Conference on Computer Graphics and Applications (Cat. No. 98EX208).

[47] Zhou X., Wang Z., Ye H., Xu C., Gao F. (2020). Ego-planner: An esdf-free gradient-based local planner for quadrotors. IEEE Robot. Autom. Lett..

[48] Felzenszwalb P.F., Huttenlocher D.P. (2012). Distance transforms of sampled functions. Theory Comput..

[49] Liu D.C., Nocedal J. (1989). On the limited memory BFGS method for large scale optimization. Math. Program..

[50] Nocedal J., Wright S.J. (1999). Numerical Optimization.

[51] Barzilai J., Borwein J.M. (1988). Two-point step size gradient methods. IMA J. Numer. Anal..

[52] Chitta S., Sucan I., Cousins S. (2012). MoveIt! [ROS Topics]. IEEE Robot. Autom. Mag..

